# Prevalence, incidence and survival of smoldering multiple myeloma in the United States

**DOI:** 10.1038/bcj.2016.100

**Published:** 2016-10-21

**Authors:** A Ravindran, A C Bartley, S J Holton, W I Gonsalves, P Kapoor, M A Siddiqui, S K Hashmi, A L Marshall, A A Ashrani, A Dispenzieri, R A Kyle, S V Rajkumar, R S Go

**Affiliations:** 1 Division of Hematology, Mayo Clinic, Rochester, MN, USA; 2 Department of Health Sciences Research, Mayo Clinic, Rochester, MN, USA; 3 Mayo Clinic Cancer Registry, Rochester, MN, USA; 4 Robert D. and Patricia E. Kern Center for The Science of Health Care Delivery, Rochester, MN, USA

Smoldering multiple myeloma (SMM) is currently defined as MM without evidence of impending (⩾60% clonal bone marrow plasma cells, serum involved to uninvolved free light chain ratio of ⩾100 with absolute involved light chain level of ⩾100 mg/L, or >1 focal lesion on magnetic resonance imaging ⩾5 mm in size) or active (hyper*c*alcemia, *r*enal insufficiency, *a*nemia or *b*one lesion – *crab* signs) end organ damage, which are considered indications for treatment.^1^ Although institutional studies show that ~8–20% of patients with MM are smoldering at the time of diagnosis,^2^ the actual prevalence of SMM in the United States (US) is unknown. Epidemiologic studies have been difficult to perform due to the lack of International Classification of Diseases (ICD) codes differentiating smoldering from active MM.

Utilizing the National Cancer Data Base (NCDB), we estimated the overall proportion of new adult MM (ICD-O: 9732) cases that were smoldering and analyzed the data according to socio-geo-demographic subgroups and type of treatment facility. We also compared the overall survival (OS) of SMM to active MM. NCDB, a joint program of the Commission on Cancer of the American College of Surgeons and the American Cancer Society, is the largest public cancer database in the US. It receives oncology outcomes data from ~1500 Commission on Cancer-accredited cancer programs covering >70% of all newly diagnosed cancer cases in the US. We included all new MM patients diagnosed from 2003 to 2011 (*N*=92 993). Follow-up data were collected until the end of 2012. NCDB records the time to initial treatment or reasons for not receiving treatment. We considered a patient to have active MM if any treatment (chemotherapy and/or radiation) was recommended by the attending physician within 120 days of MM diagnosis regardless of whether it was actually administered. Reasons for not administering treatment included co-morbidities, advanced age, patient refusal or death before treatment could be initiated. Patients who did not require treatment within 120 days of MM diagnosis were considered to have SMM. Patients with the following characteristics (due to missing data or short follow-up) were considered to have MM with unknown disease activity: (a) vital status alive or unknown and treatment not recommended but follow-up <120 days; and (b) treatment recommendation not recorded and vital status alive or unknown but with ⩾120 days of follow-up. Since the cause of death is not captured in NCDB, we considered patients who died within 120 days of MM diagnosis to have active MM even if no treatment was initially recommended as we could not exclude transformation into active MM. A detailed algorithm of disease activity classification is shown in Supplementary Figure 1. We excluded patients with missing follow-up data (*n*=157). Patients who were diagnosed (*n*=6473) but did not receive any treatment at the reporting facility (class of case 00) were excluded from the survival analysis as follow-up information might be incomplete. We analyzed the Surveillance, Epidemiology, and End Results Program (SEER) data from 2003–2011 (18 registries) using SEER*Stat software version 8.3.2 (National Cancer Institute, Bethesda, MD, USA) to determine the incidence of all MM age-adjusted to the 2000 US standard population. The incidence of SMM was then derived from this based on the proportion of MM considered to be smoldering. SEER collects cancer incidence and survival data from cancer registries covering ~28% of the US population (www.seer.cancer.gov).

Of the 86 327 MM patients included in the study, 13.7% were SMM with a median age at diagnosis of 67 years. The estimated incidence was 0.9 cases per 100 000 persons. These findings are similar to a recent Swedish population-based study, wherein 14.4% of MMs were smoldering at diagnosis with an incidence of 0.4 cases per 100 000 persons. However, the algorithm for classifying MM as active versus smoldering was not described.^3^ On the basis of these data and data from the American Cancer Society, we estimated that there will be ~4 100 cases of newly diagnosed SMM in 2016.^4^ The median age and proportion of SMM did not change significantly during the study period (*P*=0.23 and 0.34, respectively). The proportions of SMM according to socio-geo-demographic subgroups as well as co-morbidity and type of treatment facility are shown in Figure 1. The proportion of SMM was higher among those who were women, Black, older, less educated, had fewer medical co-morbidities, living closer to a treatment facility and evaluated in the Northeast. The proportions of SMM diagnosed at academic and non-academic facilities were similar. The median OS for SMM and active MM patients diagnosed in 2003–2007 were 54.8 and 28.6 months, respectively, whereas the median OS for those diagnosed in 2008–2011 were 67.1 and 40.2 months, respectively, (Figure 2a). The OS of SMM did not differ among the racial groups (Figure 2b; *P*=0.27). A recent population-based study showed that Blacks had a longer MM-specific survival compared with Whites.^5^ This was despite the fact that Blacks were less likely to receive high-dose chemotherapy and autologous stem cell transplantation in the up-front setting.^6^ The higher prevalence of SMM among Blacks may in part explain this paradox. This is in addition to the recent finding that Blacks may have a higher prevalence of more favorable cytogenetic abnormalities.^7^ Because our study included an unselected population of MM patients, the OS for active MM was lower compared with previous institutional data from the same era,^8^ although similar to SEER (data not shown). Population or institutional studies reporting OS for smoldering MM that included those who did and did not progress into active MM are not available for comparison. However, we expect patients with smoldering MM to have a lower OS compared with the general population even if they did not progress into active MM. This is because they would have co-morbidities that presented as signs or symptoms mimicking MM.

Our study included a large cohort comprised of >10 000 SMM patients with varied sociodemographic and geographic subgroups that is likely representative of the US population. However, we do acknowledge certain limitations. In 5.7% of the MM patients, there were missing data pertaining to treatment recommendation or follow-up status. Therefore, we were unable to definitely classify this subgroup of patients as active or smoldering. Nevertheless, it is reassuring that our current estimate is very similar to what was found in a recent study of the SEER-Medicare population (15.2%), in which there was not only access to the type of disease complications at the time of MM diagnosis but also the specific treatment received.^9^ This current study included only MM patients diagnosed until the year 2011, three years before the International Myeloma Working Group updated the criteria defining disease activity.^1^ We estimate that ~10–15% of SMM in our study population would be upstaged to active MM using these new criteria.^2^

Approximately one in seven patients with MM in the US is smoldering at diagnosis. The prevalence, but not the OS, of SMM varies among various sociodemographic and geographic subgroups. Epidemiologic studies in MM should take into account an estimate of those with smoldering disease when studying population disparities in treatment utilization and survival outcome. Our results can be used in the future to study the health care impact of SMM in the US.

## Figures and Tables

**Figure 1:**
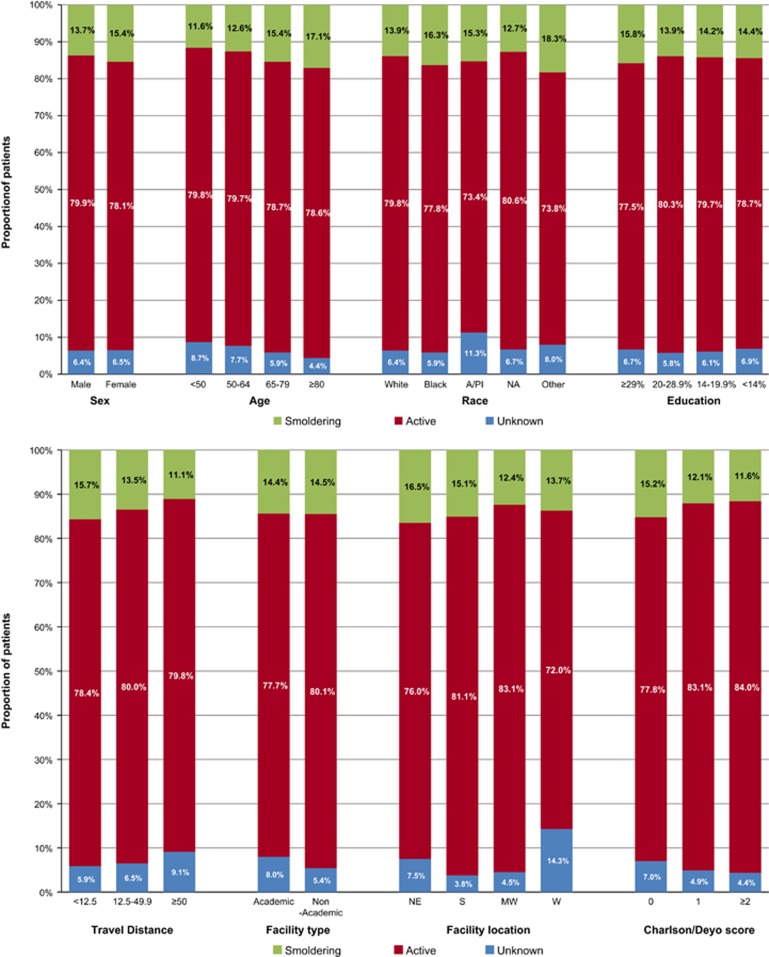
Proportion of patients with SMM according to subgroups, including sociodemographic, geographic, treatment facility and co-morbidity. A/PI, Asian/Pacific Islander; NA, Native American; MW, Midwest; NE, Northeast; S, South; W, West. The level of education was classified by quartiles of percentage of adults in the patient's zip code who did not graduate from high school. Travel distance was calculated in miles as the distance between the patient's residence and treatment facility. *P*-value was <0.01 for all comparisons within each subgroup.

**Figure 2:**
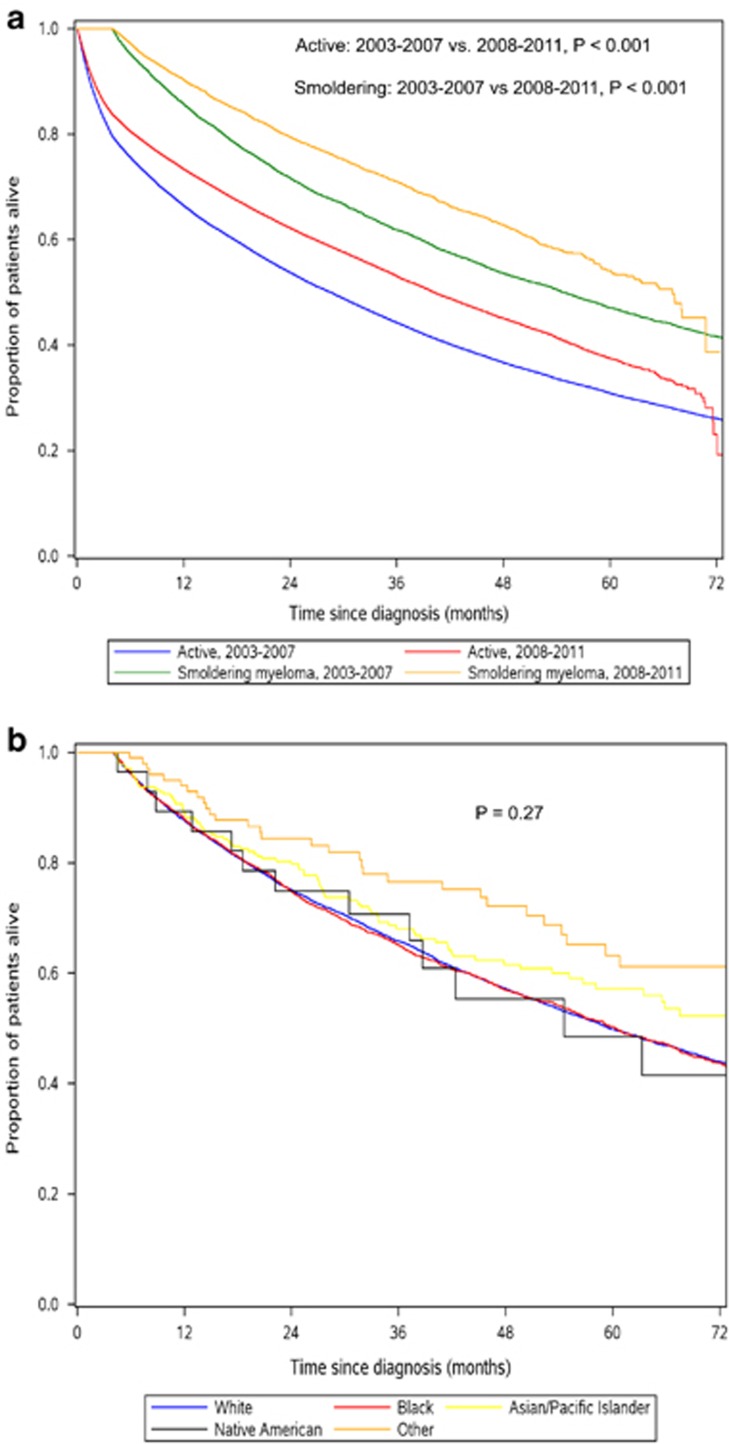
OS of multiple myeloma according to disease activity and era of diagnosis (**a**) and racial comparison of SMM survival (**b**). Survival time for smoldering myeloma started at 3 months from diagnosis, since a minimum of 120 days of follow-up was necessary to meet our definition of smoldering disease.
