# Supplementary material for: Prevalence, incidence and survival of smoldering multiple myeloma in the United States
Source: Blood Cancer J. 2016 Oct 21;6(10):e486–. doi: 10.1038/bcj.2016.100 (PMC5098258; doi:10.1038/bcj.2016.100)
Supplement: Supplementary Figure 1 [file bcj2016100x1.docx]

**Supplementary figure 1: Algorithm used in determining the disease activity of multiple myeloma patients.**
